# Classical Triad and Periventricular Lesions Do Not Necessarily Indicate Wernicke's Encephalopathy: A Case Report and Review of the Literature

**DOI:** 10.3389/fneur.2020.00451

**Published:** 2020-06-10

**Authors:** Lisha Ye, Zhouwei Xu, Jiangshan Deng, Jiajun Yang

**Affiliations:** ^1^Department of Neurology, Shanghai Jiao Tong University Affiliated Sixth People's Hospital, Shanghai, China; ^2^School of Medicine, Shanghai Jiao Tong University, Shanghai, China

**Keywords:** neuromyelitis optica, Wernicke's encephalopathy, triad, magnetic resonance imaging, periventricular lesions, differential diagnosis

## Abstract

The classical triad—ophthalmoplegia, cerebellar dysfunction, and altered mental state—in addition to bilateral symmetrical periventricular lesions are actually common to see, and clinicians tend to associate that with Wernicke's encephalopathy (WE). The diagnosis is strengthened with a likely deficiency of thiamine. We herein describe a malnourished patient with clinical triad and hyperintensities in the circumventricular regions, and she turned out to have neuromyelitis optica spectrum disorder (NMOSD) after many twists and turns. Despite totally different pathogenic mechanisms, NMOSD can mimic WE, sometimes even exhibiting radiological features similar to that of WE, thereby complicating the diagnosis. Our case highlights how similar these two diseases could be and the importance of differential diagnosis in clinical practice, which are so far rarely reported. Some clinical and radiological differences of these two diseases are summarized to help establish a prompt diagnosis.

## Background

NMOSD is a relapsing autoimmune disorder of the central nervous system (CNS), predominately affecting the optic nerve and spinal cord (1). WE is a metabolic brain disease resulting from thiamine deficiency, characterized by the triad of ophthalmoplegia, altered mental state, and trunk ataxia (2). Despite totally different pathogenic mechanisms, these two diseases have some overlaps in clinical and imaging features. However, the differential diagnosis between NMOSD and WE is overlooked in clinical settings (3). Here, we report a case in which the clinical and radiological findings misled us into the diagnosis of non-alcoholic WE before the AQP4-Ab positive result was readily available.

## Case Presentation

A 28-year-old woman was admitted to the hospital with progressive ocular symptoms as well as newly developed hemianesthesia and mental signs. Half a month before hospitalization, she complained of double eyelid droop and diplopia; the ocular weakness and fatigue was stable throughout the day and had nothing to do with physical activity. The local hospital suspected myasthenia gravis, a computed tomography scan of her brain revealed nothing, and her serum was collected to detect the acetylcholine antibody. While waiting for a hospital bed, she developed numbness in her right upper and lower extremities. At the same time, she presented with prolonged sleep duration and mental symptoms. She was then transferred to our institution. On admission, she was noted to have difficulty walking by herself due to truncal ataxia. There was no cold, fever, or diarrhea before her symptom onset.

She had been suffering from chronic anemia, and her family reported that she had dietary deficiencies due to poor appetite although she denied it herself. She was on no other medications, not pregnant, and did not use alcohol or illicit drugs.

On physical examination, the patient presented obviously unbalanced nutrition; she was 1.65 m in height yet only weighed 40 kg. She was conscious and oriented and scored 23 in the MMSE, mainly due to impaired memory and calculation performance. Eye signs were obvious; she had drooping eyelids (both 5 mm). The ophthalmoplegia was remarkable; she demonstrated bilateral abducens nerve palsies and adduction deficit of both eyes. Limitation and nystagmus on vertical gaze were also noted. There was slight dysmetria on both finger–nose and heel–shin testing. Limb dysdiadochokinesia was also observed. Bilateral pyramidal signs were positive. Sensory examination indicated hyperesthesia on the right side. Findings on muscle tone and strength tests were normal.

She had an abnormal Hb level of 92 g/L, accompanied by low levels of serum folate and ferritin; vitamin B12 level was normal. Laboratory tests also revealed elevated levels of CA199 and CA242; both were gastrointestinal tumor markers although an abdominal enhanced CT scan reported no findings. The CSF revealed an increased white blood cell count of 28/mL (dominantly lymphocytes); other routine analyses of the cerebrospinal fluid (CSF) were normal, including protein level and IgG index (0.49). Also, no oligoclonal bands were found. Additional laboratory workup—including rheumatology analysis—was negative.

**Figure 1:**
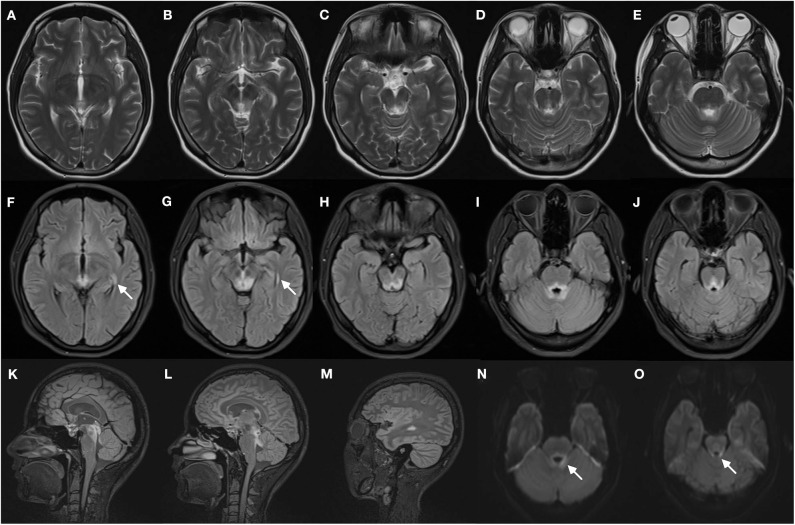
Brain magnetic resonance images of the patient. T2-weighted images **(A–E)** demonstrate bilateral symmetrical hyperintense lesions in periaqueductal regions and around the fourth ventricle, which include superior colliculus **(A)**, ventral tegmental area **(B)**, inferior colliculus **(C)**, and superior cerebellar peduncle **(D,E)**. The abnormal regions revealed more obvious hyperintensity signal on corresponding FLAIR images **(F–J)**, increased signal in the lateral ventricles was found (**F,G**, arrows). The FLAIR sagittal slice showed apparent hyperintense signal in the periaqueductal gray region **(K)** and surrounding the fourth ventricle **(L)**, and lesions in the lateral ventricles **(M)**. DWI images also manifest restricted water diffusion adjacent to the fourth ventricle (**N,O**, arrows).

Magnetic resonance imaging of the brain (Figure 1) showed symmetric T2 hyperintensities predominantly located within periaqueductal gray matter and periventricular regions of the fourth ventricles. A diffusion-weighted sequence also showed a hyperintense signal along the floor of the fourth ventricle. The presence of a lateral ventricle lesion was also noted. All brain lesions did not show contrast enhancement. Nerve conduction studies and visual evoked potentials were normal.

In view of the possible diagnosis of WE, thiamine was started intramuscularly (300 mg 12-h). However, poor clinical recovery was observed. Her mental status worsened and exhibited hallucination, and the ocular palsy also progressed.

**Figure 2:**
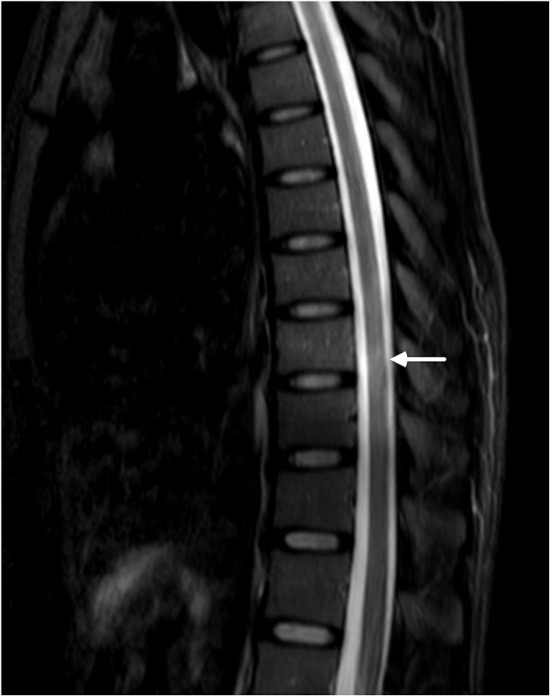
MRI of the lower thoracic spine showed T2 hyperintense lesions from T7 through T9 (arrow). However, the signal was blurry, and we considered it to be undetermined.

Then came the crucial result that her serum and cerebral fluid tested positive for NMOSD-IgG (1:32 and 1:3.2, respectively). Thereafter, the spinal cord was evaluated, and MRI of the spine revealed a suspicious enhanced T2-weighted signal at the lower thoracic cord region (Figure 2).

We corrected the diagnosis, and the patient started with treatment of high-dose intravenous steroids, but still little improvement was detected. The patient then experienced bilateral visual loss, but MRI results of the optic nerve reported no findings. After that, intravenous immunoglobulin treatment was given, followed by intravenous cyclophosphamide therapy. On discharge, she showed moderate improvement in eye movement and ataxia although her vision remained unchanged.

## Discussion

In our case, the patient presented the classical triad of ocular signs, mental status changes, and unsteadiness of gait; even the MRI lesion patterns perfectly corresponded to the diagnosis of non-alcoholic WE. Underlying digestive tract cancer (clued by her chronic anemia and elevated tumor markers) was suspected to be responsible for the malabsorption of vitamin B1, making the diagnosis fairly reassuring. But there were some discoveries that did not match the diagnosis. First, the pleocytosis in CSF was incompatible with a WE diagnosis. Second, the patient didn't achieve the desired therapeutic effect. WE patients are reported to have good and even dramatic responses to thiamine treatment, and in our case, very little recovery occurred despite adequate supplementation with thiamine.

Interestingly, WE and NMOSD both feature periependymal lesions surrounding the ventricular system, thus sharing some clinical and imaging features. Contrasting different pathogenic processes of these two diseases may help us to better understand such unique brain abnormalities. For NMOSD seropositive for anti-AQP4 antibody, the pathogenesis is initiated by the specific autoantibody AQP4-IgG targeting the autoantigen, aquaporin-4, thus particularly demonstrating abnormalities in brain regions with high AQP4 expression, that is, pial and ependymal surfaces in contact with the CSF (4). The entry of AQP4–IgG into the CNS and its binding inhibits regional AQP4 water permeability, causing astrocyte swelling (5). The exact mechanism that accounts for the typical lesion pattern of WE remains unclear. One possible hypothesis is that thiamine deficiency results in insufficient production of thiamine pyrophosphate (6), which is an important coenzyme to process carbohydrate metabolism in the brain (7). As a result of energy metabolism dysfunction, lactic acid starts to accumulate in the focal regions of brain. And it is reported that lactic acidosis may mediate AQP-4 overexpression in astrocytes, leading to cytotoxic edema (8). Once again, AQP-4 is particularly concentrated on circumventricular areas (4), which are also selective histological lesion locations for WE.

At present, the diagnosis of WE is primarily clinical as thiamine measurement is often unavailable in many hospitals, and no specific abnormalities have been identified in other laboratory tests (9). MRI also functions as a supportive diagnostic adjunct. Although it is reported that MRI has a high specificity of 93% for the diagnosis of WE (10), we should not forget that such brain lesions are also typical patterns of NMOSD. NMOSD has variable clinical presentations; involvement of brainstem and diencephalon could cause the clinical triad of ophthalmoplegia, ataxia, and confusion, all of which can be seen in WE. And we particularly have to point out that visual loss is not exclusive for NMOSD, WE can also manifest as visual deterioration, which is often resulted from optic disk swelling (11) but in relatively low incidence.

The differential diagnosis between WE and NMO tends to be overlooked and possibly underreported, which may lead to an incorrect or delayed diagnosis. In a case reported by Nyo et al. (12), a young woman with SLE developed the clinical triad of mild confusion, ataxia, and ophthalmoplegia; hyperintensity in the thalami and cerebellar of MRI was noticed, and then she was diagnosed as WE secondary to a likely gastrointestinal SLE. After treating with intravenous methylprednisolone and oral prednisone, the patient had a full neurological recovery. However, in our opinion, the possibility of NMOSD should be considered for the following reasons: (1) She was a young woman, (2) she had a medical history of SLE, (3) the symptoms of pancerebellar ataxia and dysarthria were unusual for WE, (4) she had raised protein concentrations in CSF, (5) she had indefinite thiamine deficiency, and (6) effective treatment of cortisol was combined with thiamine. Long-term follow-up is highly recommended for this person, and AQP4-Ab should be detected if possible.

Its extremely important to differentiate WE from NMOSD for they are distinct in treatment. NMOSD requires immunosuppressive therapy, and glucocorticoid is effective at reducing inflammation (13). Although WE requires emergent thiamine supplementation, the management of glucocorticoid should be cautious because exact effects of steroid treatment in WE are unclear.

We summarize features that differ between NMOSD and WE in Table 1; meanwhile, some important points are highlighted below.

**Table 1:** Distinctive characteristics of NMOSD from those of WE.

		**WE**		**NMOSD**
**Epidemiology**				
Prevalence		0.4–2.8% based on autopsies findings (14), higher in alcoholics		Inconsistent findings, varies from 2 to 100 per million people, relatively common in non-Caucasian race (15)
female-to-male ratio		Around 1:1.7 (9)		Female prominence (3:1–9:1) (16)
Age of onset		Vary greatly on age (9)		Median age of 39 years old (1)
**Medical history**		Thiamine depletion (see point i), mostly due to alcohol abuse (17)		Often associated with systemic autoimmune disease (18)
**Symptoms and signs**		Alcoholics (17)	Non-alcoholics (17)	
Brain	Ocular abnormalities	90.2%	70.7%	Relatively uncommon
	Mental status changes	75.5%	67.2%	35–67% (19)
	Trunk ataxia	82%	58%	Relatively uncommon
	Nausea and vomiting	6.9%	31%	16–43% (20)
	Seizure	None	None	Uncommon
	Triad	53.9%	33.6%	Relatively uncommon
Myelitis		None		Common, 30–70% at first attack (21)
Visual impairment		Rare		Common (50%) (22)
**MRI findings**				
Brain	Periventricular lesions	Common, around 53% (10)		Common, 12–40% (23)
	Hyperintensity of mammillary bodies	Common, sometimes the only sign (17)		Rare
	Hemispheric white matter lesions.	Unknown		Common
	Dots or patches of hyperintensities	Unknown		Non-specific, 35–84% (23)
Myelitis		None		Longitudinally extensive lesion (23)
Optic nerve		Rare		Long-length/posterior-chiasmal lesions (23)
**Serology**				
Thiamine detection		Reduced level of thiamine		Normal
Serum AQP4-Ab		Not present		Present in average of 72% of NMO (24)
**Cerebrospinal fluid study**				
Pleocytosis		Rare		mildly elevated, median 19/μl during acute attack (25)
Protein level		Usually normal, moderate elevation in late stage (2)		Elevated protein level (up to 50% of patients) (25)
**Prognosis**				
Clinical course		Progressive, if untreated		Can be recurrent
Mortality		17% (14)		Ranges from 2.9 to 25% (15)

*AQP4-ab, autoantibody to aquaporin-4; WE, Wernicke's encephalopathy; NMOSD, Neuromyelitis optica spectrum disorder*.

### Thiamine Deficiency

A history of thiamine depletion is necessary before reaching a diagnosis of WE, and it has been emphasized as the fourth sign apart from the triad in the revised criteria (17). If commercially available, measurement of blood thiamine or its phosphate ester concentrations should be performed to confirm the thiamine deficiency. Although alcohol abuse accounts for most cases, non-alcoholic WE has also been frequently reported, and according to Galvin et al. (17), the most common conditions are tumors, gastrointestinal disease, and surgery as well as hyperemesis gravidarum. Other causes reported include starvation, dieting, and unbalanced diets. Symptoms such as wet beriberi or polyneuropathy also hint at thiamine insufficiency (26). However, we sometimes overestimate the unbalanced nutrition or a potential malignant disorder as shown in our case. Still, any patient suspected of having WE should be treated with a high dose of thiamine supplementation immediately, considering the significant mortality and neurological disabilities. Also, recovery of the neurological state after administration of thiamine is valuable for diagnosis (9).

### CSF Assay

Lumbar puncture should be done on every suspected WE. CSF testing is normal in most WE patients although increased protein levels in late stages were occasionally reported (2). Moderate pleocytosis in the CSF is often a feature of NMOSD (25), which is rare to see in WE.

### Brain MRI Differences

Distinguishing WE from NMOSD can be tough when they manifest a similar altered signal in lesions around the third ventricles, adjacent to the fourth ventricle, or surrounding the lateral ventricles. Thalamus, hypothalamus, and brainstem are also susceptible in both diseases. But, generally speaking, brain lesions in the mammillary bodies favor the diagnosis of WE (27), whereas NMOSD frequently features abnormalities in hemispheric white matter and optic nerve lesions (28).

### Spinal Cord Involvement

To our knowledge, WE selectively involves cerebral, and there is no spinal cord engagement. Therefore, thorough investigations in search of an underlying sensory level is warranted when making differential diagnoses. Also, in patients suspected to have NMOSD, spinal cord MRI should be performed.

### AQP4-IgG

The AQP4-Ab assay should be tested for suspected NMOSD, and sometimes, the presence of this specific marker can play a determinant role in distinguishing these diseases. However, the absence of AQP4-IgG doesn't exclude the possibility of NMOSD (29).

## Conclusion

The overlaps in phenotypes between WE and NMOSD might obscure the correct diagnosis. Our case highlights that, for those presenting with typical triad and circumventricular lesions, NMOSD should not be neglected as an important differential diagnosis, especially for young women. Some clinical and neuroimaging evidence can help distinguish NMOSD from WE. Prompt recognition and proper treatment can prevent neurological deterioration and improve the outcome. Still, patients suspected to have WE should be empirically treated with thiamine promptly.

## Data Availability Statement

All datasets generated for this study are included in the article/supplementary material.

## Ethics Statement

The studies involving human participants were reviewed and approved by Shanghai sixth hospital ethics committee. The patients/participants provided their written informed consent to participate in this study. Written informed consent was obtained from the individual(s) for the publication of any potentially identifiable images or data included in this article.

## Author Contributions

LY drafted the manuscript. ZX, JD, and JY critically reviewed the manuscript.

## Conflict of Interest

The authors declare that the research was conducted in the absence of any commercial or financial relationships that could be construed as a potential conflict of interest.
